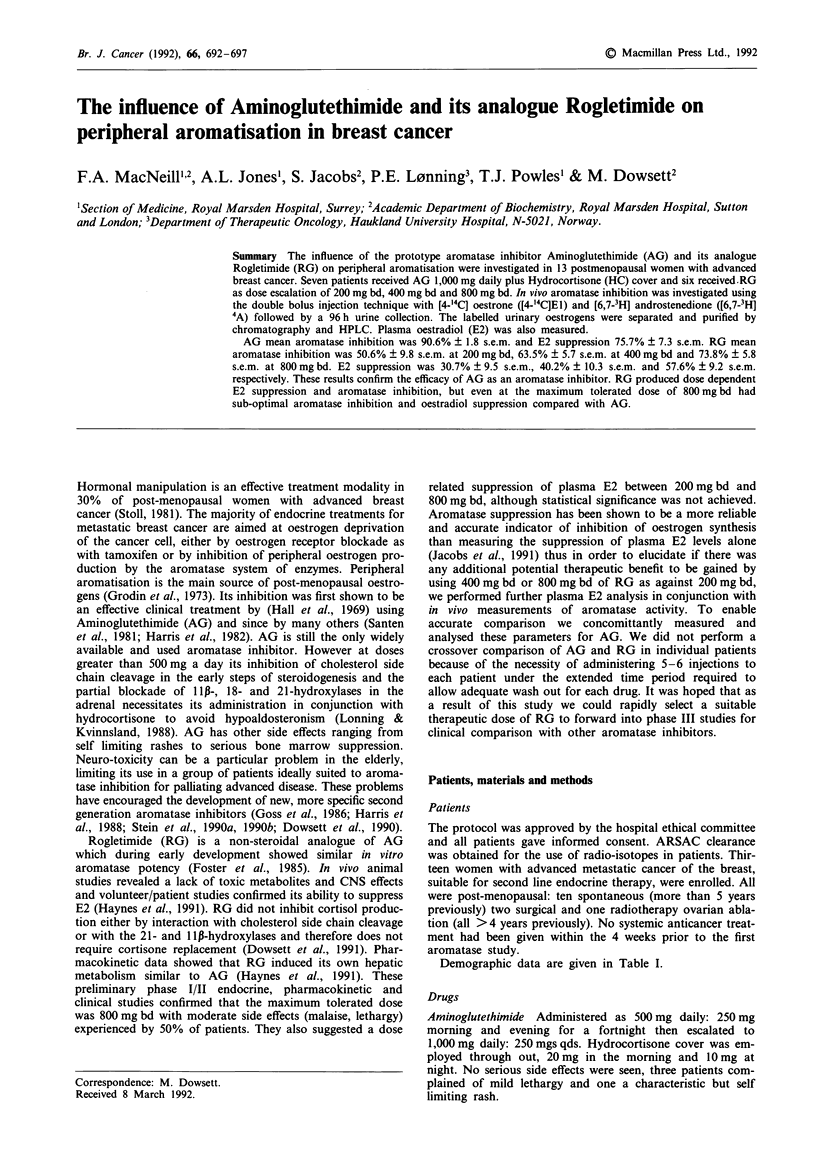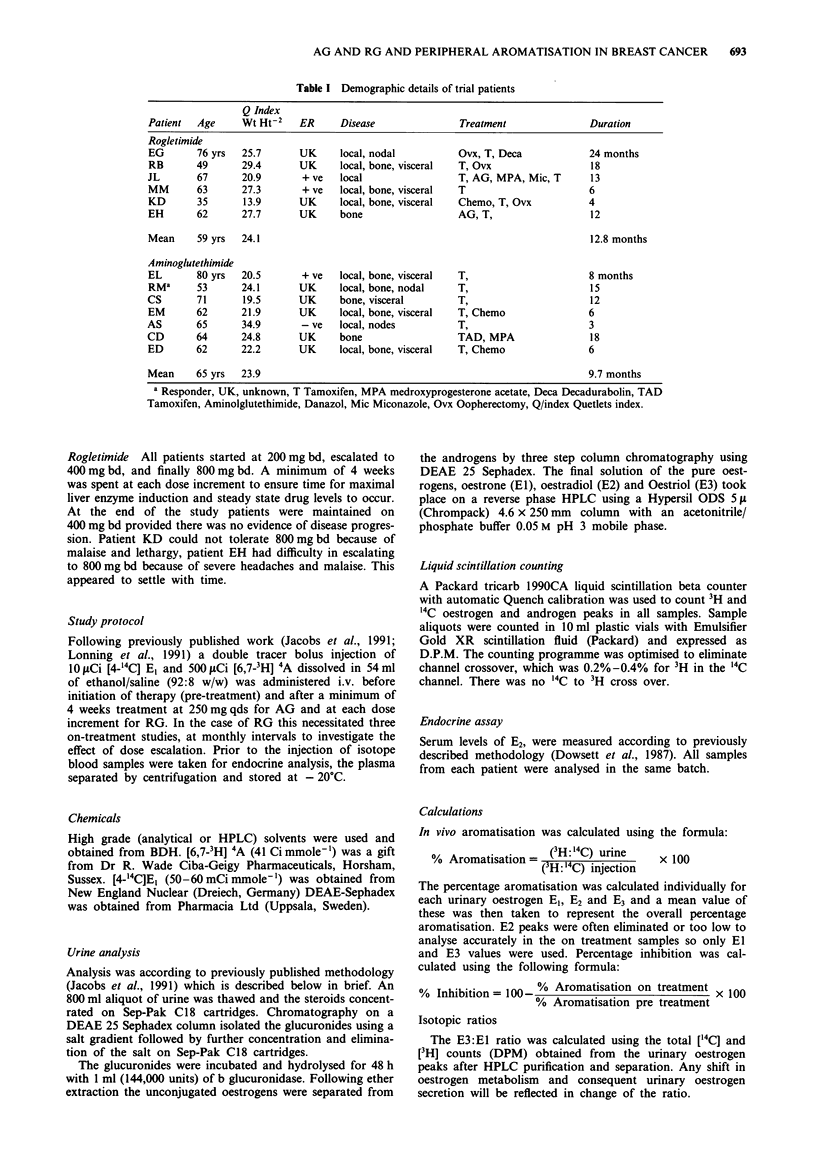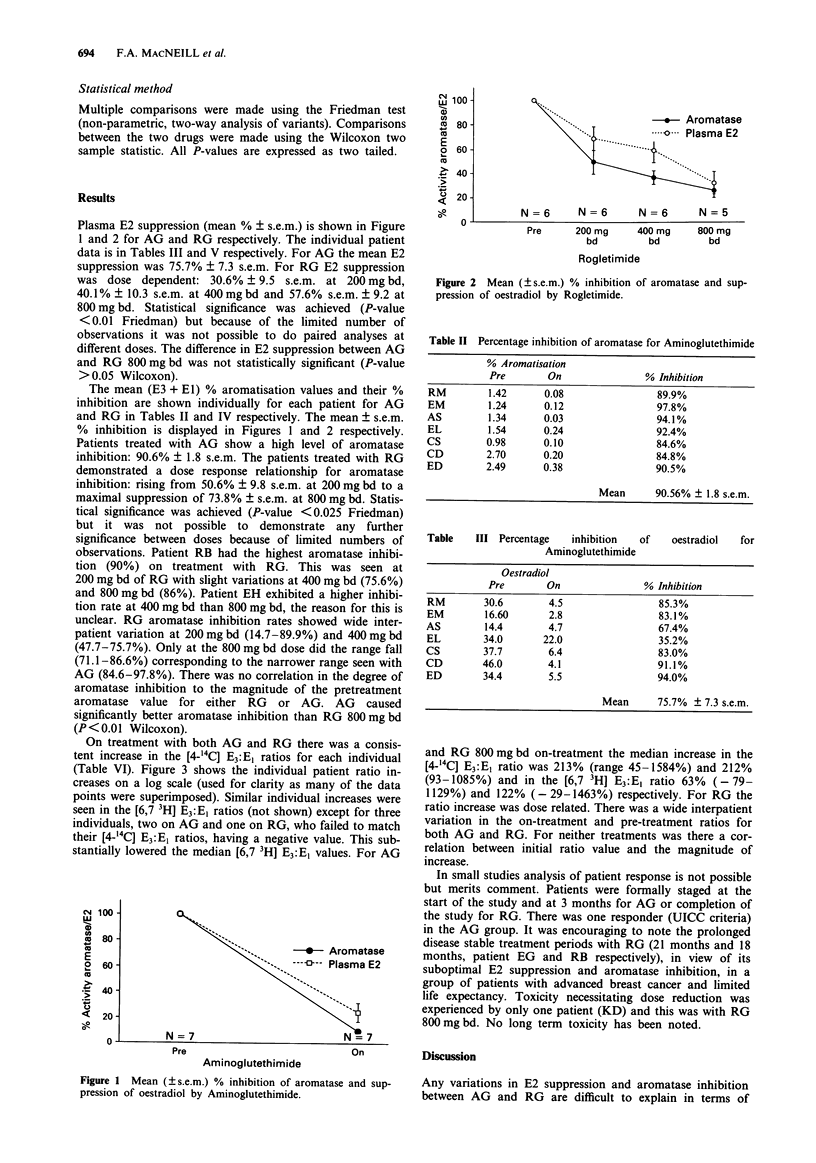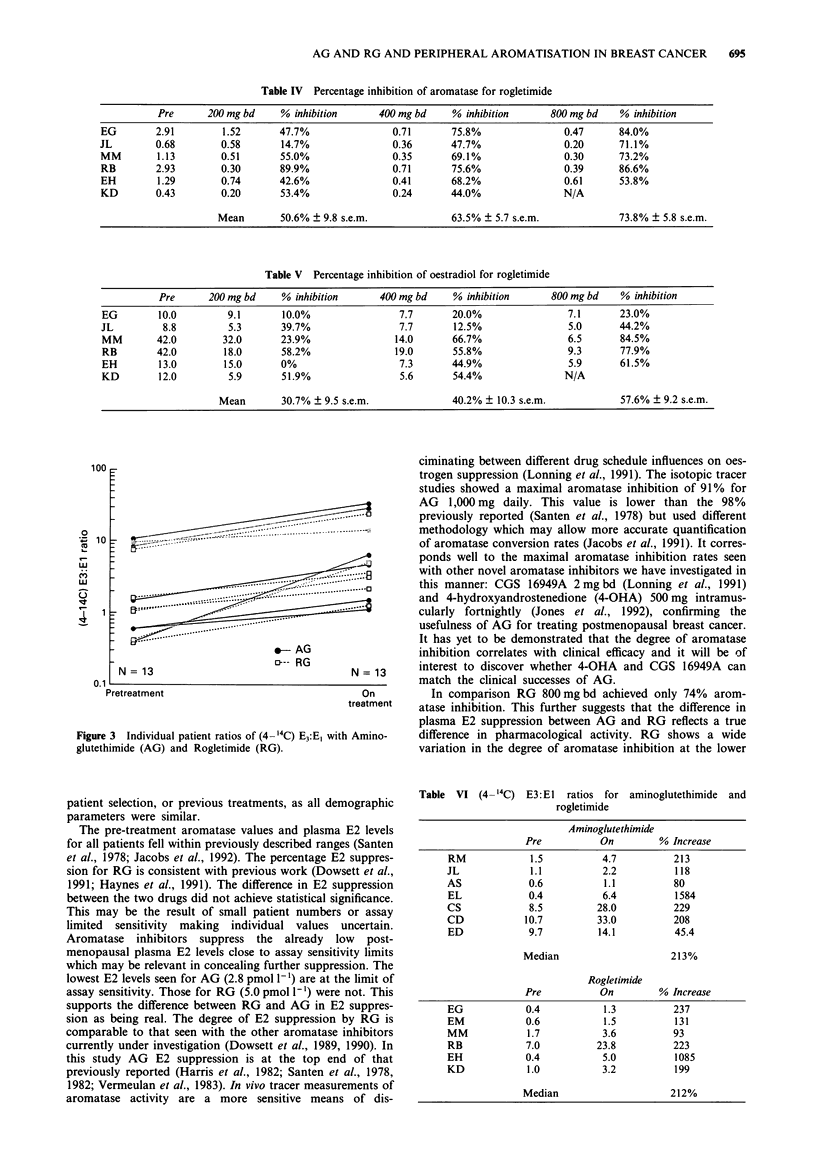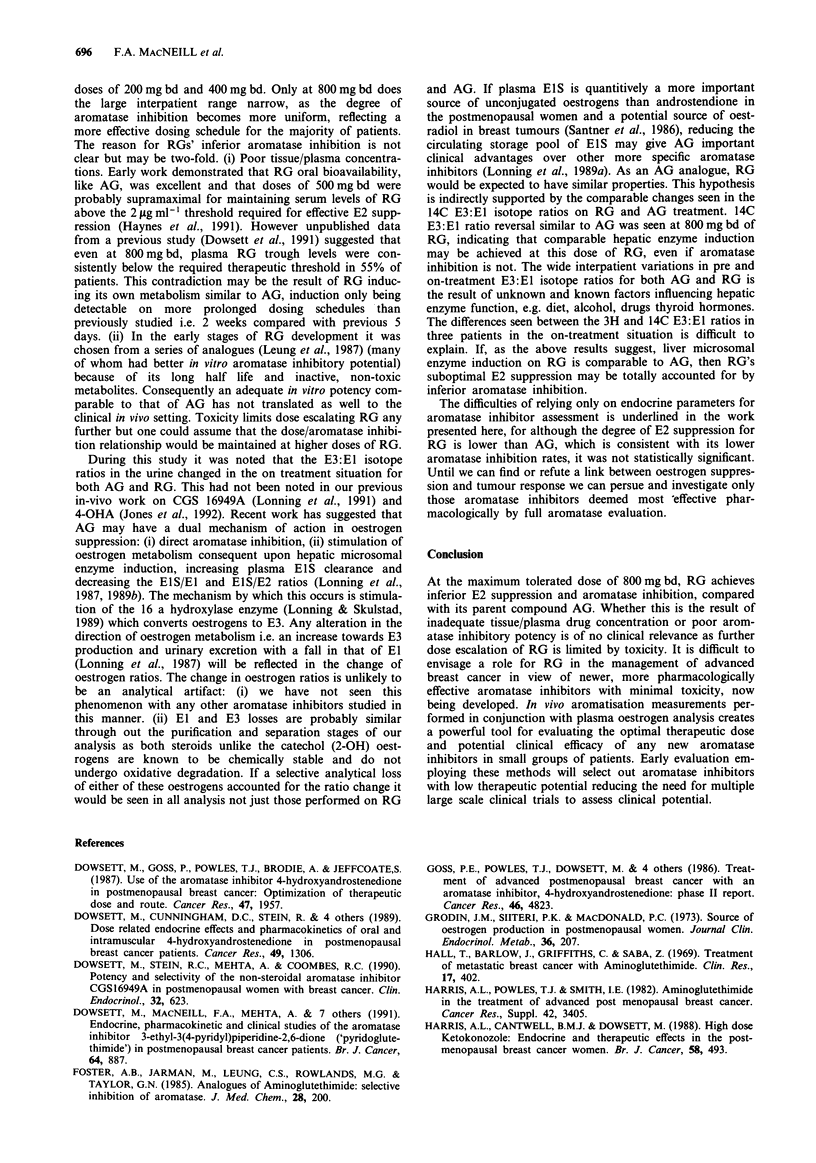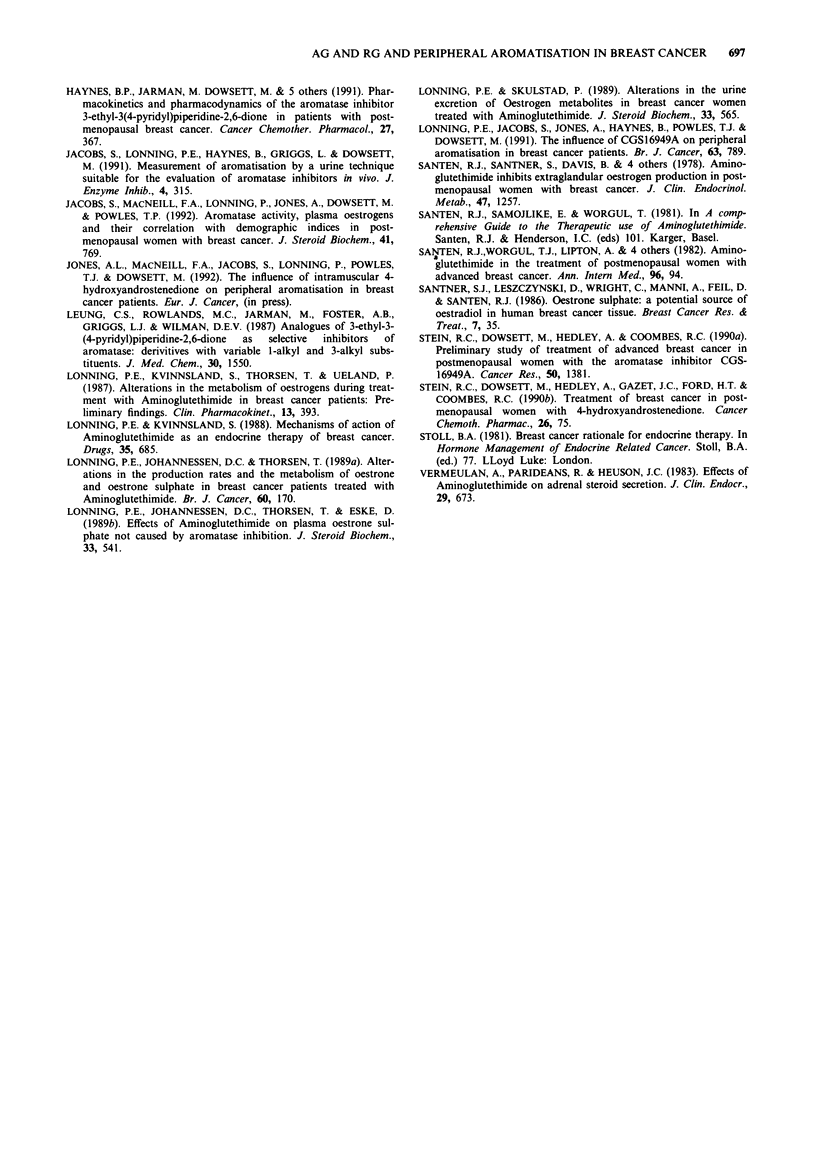# The influence of aminoglutethimide and its analogue rogletimide on peripheral aromatisation in breast cancer.

**DOI:** 10.1038/bjc.1992.339

**Published:** 1992-10

**Authors:** F. A. MacNeill, A. L. Jones, S. Jacobs, P. E. Lønning, T. J. Powles, M. Dowsett

**Affiliations:** Section of Medicine, Royal Marsden Hospital, Surrey.

## Abstract

The influence of the prototype aromatase inhibitor Aminoglutethimide (AG) and its analogue Rogletimide (RG) on peripheral aromatisation were investigated in 13 postmenopausal women with advanced breast cancer. Seven patients received AG 1,000 mg daily plus Hydrocortisone (HC) cover and six received RG as dose escalation of 200 mg bd, 400 mg bd and 800 mg bd. In vivo aromatase inhibition was investigated using the double bolus injection technique with [4-14C] oestrone ([4-14C]E1) and [6,7-3H] androstenedione ([6,7-3H]4A) followed by a 96 h urine collection. The labelled urinary oestrogens were separated and purified by chromatography and HPLC. Plasma oestradiol (E2) was also measured. AG mean aromatase inhibition was 90.6% +/- 1.8 s.e.m. and E2 suppression 75.7% +/- 7.3 s.e.m. RG mean aromatase inhibition was 50.6% +/- 9.8 s.e.m. at 200 mg bd, 63.5% +/- 5.7 s.e.m. at 400 mg bd and 73.8% +/- 5.8 s.e.m. at 800 mg bd. E2 suppression was 30.7% +/- 9.5 s.e.m., 40.2% +/- 10.3 s.e.m. and 57.6% +/- 9.2 s.e.m. respectively. These results confirm the efficacy of AG as an aromatase inhibitor. RG produced dose dependent E2 suppression and aromatase inhibition, but even at the maximum tolerated dose of 800 mg bd had sub-optimal aromatase inhibition and oestradiol suppression compared with AG.


Br. J. Cancer (1992), 66, 692-697 © Macmillan Press Ltd., 1992

### The influence of Aminoglutethimide and its analogue Rogletimide on peripheral aromatisation in breast cancer

F.A. MacNeill[1,2], A.L. Jones[1], S. Jacobs[2], P.E. Lønning[3], T.J. Powles[1] & M. Dowsett[2]

[1]*Section of Medicine, Royal Marsden Hospital, Surrey;* [2]*Academic Department of Biochemistry, Royal Marsden Hospital, Sutton and London;* [3]*Department of Therapeutic Oncology, Haukland University Hospital, N-5021, Norway.*

**Summary** The influence of the prototype aromatase inhibitor Aminoglutethimide (AG) and its analogue Rogletimide (RG) on peripheral aromatisation were investigated in 13 postmenopausal women with advanced breast cancer. Seven patients received AG 1,000 mg daily plus Hydrocortisone (HC) cover and six received RG as dose escalation of 200 mg bd, 400 mg bd and 800 mg bd. *In vivo* aromatase inhibition was investigated using the double bolus injection technique with [4-$^{14}$C] oestrone ([4-$^{14}$C]E1) and [6,7-$^{3}$H] androstenedione ([6,7-$^{3}$H] $^{4}$A) followed by a 96 h urine collection. The labelled urinary oestrogens were separated and purified by chromatography and HPLC. Plasma oestradiol (E2) was also measured.

AG mean aromatase inhibition was 90.6% ± 1.8 s.e.m. and E2 suppression 75.7% ± 7.3 s.e.m. RG mean aromatase inhibition was 50.6% ± 9.8 s.e.m. at 200 mg bd, 63.5% ± 5.7 s.e.m. at 400 mg bd and 73.8% ± 5.8 s.e.m. at 800 mg bd. E2 suppression was 30.7% ± 9.5 s.e.m., 40.2% ± 10.3 s.e.m. and 57.6% ± 9.2 s.e.m. respectively. These results confirm the efficacy of AG as an aromatase inhibitor. RG produced dose dependent E2 suppression and aromatase inhibition, but even at the maximum tolerated dose of 800 mg bd had sub-optimal aromatase inhibition and oestradiol suppression compared with AG.

Hormonal manipulation is an effective treatment modality in 30% of post-menopausal women with advanced breast cancer (Stoll, 1981). The majority of endocrine treatments for metastatic breast cancer are aimed at oestrogen deprivation of the cancer cell, either by oestrogen receptor blockade as with tamoxifen or by inhibition of peripheral oestrogen production by the aromatase system of enzymes. Peripheral aromatisation is the main source of post-menopausal oestrogens (Grodin *et al.*, 1973). Its inhibition was first shown to be an effective clinical treatment by (Hall *et al.*, 1969) using Aminoglutethimide (AG) and since by many others (Santen *et al.*, 1981; Harris *et al.*, 1982). AG is still the only widely available and used aromatase inhibitor. However at doses greater than 500 mg a day its inhibition of cholesterol side chain cleavage in the early steps of steroidogenesis and the partial blockade of 11β-, 18- and 21-hydroxylases in the adrenal necessitates its administration in conjunction with hydrocortisone to avoid hypoaldosteronism (Lonning & Kvinnsland, 1988). AG has other side effects ranging from self limiting rashes to serious bone marrow suppression. Neuro-toxicity can be a particular problem in the elderly, limiting its use in a group of patients ideally suited to aromatase inhibition for palliating advanced disease. These problems have encouraged the development of new, more specific second generation aromatase inhibitors (Goss *et al.*, 1986; Harris *et al.*, 1988; Stein *et al.*, 1990*a*, 1990*b*; Dowsett *et al.*, 1990).

Rogletimide (RG) is a non-steroidal analogue of AG which during early development showed similar *in vitro* aromatase potency (Foster *et al.*, 1985). *In vivo* animal studies revealed a lack of toxic metabolites and CNS effects and volunteer/patient studies confirmed its ability to suppress E2 (Haynes *et al.*, 1991). RG did not inhibit cortisol production either by interaction with cholesterol side chain cleavage or with the 21- and 11β-hydroxylases and therefore does not require cortisone replacement (Dowsett *et al.*, 1991). Pharmacokinetic data showed that RG induced its own hepatic metabolism similar to AG (Haynes *et al.*, 1991). These preliminary phase I/II endocrine, pharmacokinetic and clinical studies confirmed that the maximum tolerated dose was 800 mg bd with moderate side effects (malaise, lethargy) experienced by 50% of patients. They also suggested a dose related suppression of plasma E2 between 200 mg bd and 800 mg bd, although statistical significance was not achieved. Aromatase suppression has been shown to be a more reliable and accurate indicator of inhibition of oestrogen synthesis than measuring the suppression of plasma E2 levels alone (Jacobs *et al.*, 1991) thus in order to elucidate if there was any additional potential therapeutic benefit to be gained by using 400 mg bd or 800 mg bd of RG as against 200 mg bd, we performed further plasma E2 analysis in conjunction with *in vivo* measurements of aromatase activity. To enable accurate comparison we concomittantly measured and analysed these parameters for AG. We did not perform a crossover comparison of AG and RG in individual patients because of the necessity of administering 5-6 injections to each patient under the extended time period required to allow adequate wash out for each drug. It was hoped that as a result of this study we could rapidly select a suitable therapeutic dose of RG to forward into phase III studies for clinical comparison with other aromatase inhibitors.

Correspondence: M. Dowsett.
Received 8 March 1992.

### Patients, materials and methods

### *Patients*

The protocol was approved by the hospital ethical committee and all patients gave informed consent. ARSAC clearance was obtained for the use of radio-isotopes in patients. Thirteen women with advanced metastatic cancer of the breast, suitable for second line endocrine therapy, were enrolled. All were post-menopausal: ten spontaneous (more than 5 years previously) two surgical and one radiotherapy ovarian ablation (all >4 years previously). No systemic anticancer treatment had been given within the 4 weeks prior to the first aromatase study.

Demographic data are given in Table I.

### *Drugs*

*Aminoglutethimide* Administered as 500 mg daily: 250 mg morning and evening for a fortnight then escalated to 1,000 mg daily: 250 mgs qds. Hydrocortisone cover was employed through out, 20 mg in the morning and 10 mg at night. No serious side effects were seen, three patients complained of mild lethargy and one a characteristic but self limiting rash.

**Table I** Demographic details of trial patients

| *Patient* | *Age* | *Q Index* Wt Ht$^{-2}$ | *ER* | *Disease* | *Treatment* | *Duration* |
|---|---|---|---|---|---|---|
| *Rogletimide* | | | | | | |
| EG | 76 yrs | 25.7 | UK | local, nodal | Ovx, T, Deca | 24 months |
| RB | 49 | 29.4 | UK | local, bone, visceral | T, Ovx | 18 |
| JL | 67 | 20.9 | + ve | local | T, AG, MPA, Mic, T | 13 |
| MM | 63 | 27.3 | + ve | local, bone, visceral | T | 6 |
| KD | 35 | 13.9 | UK | local, bone, visceral | Chemo, T, Ovx | 4 |
| EH | 62 | 27.7 | UK | bone | AG, T, | 12 |
| Mean | 59 yrs | 24.1 | | | | 12.8 months |
| *Aminoglutethimide* | | | | | | |
| EL | 80 yrs | 20.5 | + ve | local, bone, visceral | T, | 8 months |
| RM[a] | 53 | 24.1 | UK | local, bone, nodal | T, | 15 |
| CS | 71 | 19.5 | UK | bone, visceral | T, | 12 |
| EM | 62 | 21.9 | UK | local, bone, visceral | T, Chemo | 6 |
| AS | 65 | 34.9 | – ve | local, nodes | T, | 3 |
| CD | 64 | 24.8 | UK | bone | TAD, MPA | 18 |
| ED | 62 | 22.2 | UK | local, bone, visceral | T, Chemo | 6 |
| Mean | 65 yrs | 23.9 | | | | 9.7 months |

[a] Responder, UK, unknown, T Tamoxifen, MPA medroxyprogesterone acetate, Deca Decadurabolin, TAD Tamoxifen, Aminolglutethimide, Danazol, Mic Miconazole, Ovx Oopherectomy, Q/index Quetlets index.

*Rogletimide* All patients started at 200 mg bd, escalated to 400 mg bd, and finally 800 mg bd. A minimum of 4 weeks was spent at each dose increment to ensure time for maximal liver enzyme induction and steady state drug levels to occur. At the end of the study patients were maintained on 400 mg bd provided there was no evidence of disease progression. Patient KD could not tolerate 800 mg bd because of malaise and lethargy, patient EH had difficulty in escalating to 800 mg bd because of severe headaches and malaise. This appeared to settle with time.

### *Study protocol*

Following previously published work (Jacobs *et al.*, 1991; Lonning *et al.*, 1991) a double tracer bolus injection of 10 $\mu$Ci [4-$^{14}$C] $E_1$ and 500 $\mu$Ci [6,7-$^3$H] $^4$A dissolved in 54 ml of ethanol/saline (92:8 w/w) was administered i.v. before initiation of therapy (pre-treatment) and after a minimum of 4 weeks treatment at 250 mg qds for AG and at each dose increment for RG. In the case of RG this necessitated three on-treatment studies, at monthly intervals to investigate the effect of dose escalation. Prior to the injection of isotope blood samples were taken for endocrine analysis, the plasma separated by centrifugation and stored at – 20°C.

### *Chemicals*

High grade (analytical or HPLC) solvents were used and obtained from BDH. [6,7-$^3$H] $^4$A (41 Ci mmole$^{-1}$) was a gift from Dr R. Wade Ciba-Geigy Pharmaceuticals, Horsham, Sussex. [4-$^{14}$C]$E_1$ (50–60 mCi mmole$^{-1}$) was obtained from New England Nuclear (Dreiech, Germany) DEAE-Sephadex was obtained from Pharmacia Ltd (Uppsala, Sweden).

### *Urine analysis*

Analysis was according to previously published methodology (Jacobs *et al.*, 1991) which is described below in brief. An 800 ml aliquot of urine was thawed and the steroids concentrated on Sep-Pak C18 cartridges. Chromatography on a DEAE 25 Sephadex column isolated the glucuronides using a salt gradient followed by further concentration and elimination of the salt on Sep-Pak C18 cartridges.

The glucuronides were incubated and hydrolysed for 48 h with 1 ml (144,000 units) of b glucuronidase. Following ether extraction the unconjugated oestrogens were separated from the androgens by three step column chromatography using DEAE 25 Sephadex. The final solution of the pure oestrogens, oestrone (E1), oestradiol (E2) and Oestriol (E3) took place on a reverse phase HPLC using a Hypersil ODS 5 $\mu$ (Chrompack) 4.6 × 250 mm column with an acetonitrile/phosphate buffer 0.05 M pH 3 mobile phase.

### *Liquid scintillation counting*

A Packard tricarb 1990CA liquid scintillation beta counter with automatic Quench calibration was used to count $^3$H and $^{14}$C oestrogen and androgen peaks in all samples. Sample aliquots were counted in 10 ml plastic vials with Emulsifier Gold XR scintillation fluid (Packard) and expressed as D.P.M. The counting programme was optimised to eliminate channel crossover, which was 0.2%–0.4% for $^3$H in the $^{14}$C channel. There was no $^{14}$C to $^3$H cross over.

### *Endocrine assay*

Serum levels of $E_2$, were measured according to previously described methodology (Dowsett *et al.*, 1987). All samples from each patient were analysed in the same batch.

### *Calculations*

*In vivo* aromatisation was calculated using the formula:

$$\%\ \text{Aromatisation} = \frac{(^3\text{H}:^{14}\text{C})\ \text{urine}}{(^3\text{H}:^{14}\text{C})\ \text{injection}} \times 100$$

The percentage aromatisation was calculated individually for each urinary oestrogen $E_1$, $E_2$ and $E_3$ and a mean value of these was then taken to represent the overall percentage aromatisation. E2 peaks were often eliminated or too low to analyse accurately in the on treatment samples so only E1 and E3 values were used. Percentage inhibition was calculated using the following formula:

$$\%\ \text{Inhibition} = 100 - \frac{\%\ \text{Aromatisation on treatment}}{\%\ \text{Aromatisation pre treatment}} \times 100$$

Isotopic ratios

The E3:E1 ratio was calculated using the total [$^{14}$C] and [$^3$H] counts (DPM) obtained from the urinary oestrogen peaks after HPLC purification and separation. Any shift in oestrogen metabolism and consequent urinary oestrogen secretion will be reflected in change of the ratio.

*Statistical method*

Multiple comparisons were made using the Friedman test (non-parametric, two-way analysis of variants). Comparisons between the two drugs were made using the Wilcoxon two sample statistic. All $P$-values are expressed as two tailed.

### Results

Plasma E2 suppression (mean % ± s.e.m.) is shown in Figure 1 and 2 for AG and RG respectively. The individual patient data is in Tables III and V respectively. For AG the mean E2 suppression was 75.7% ± 7.3 s.e.m. For RG E2 suppression was dose dependent: 30.6% ± 9.5 s.e.m. at 200 mg bd, 40.1% ± 10.3 s.e.m. at 400 mg bd and 57.6% s.e.m. ± 9.2 at 800 mg bd. Statistical significance was achieved ($P$-value $<0.01$ Friedman) but because of the limited number of observations it was not possible to do paired analyses at different doses. The difference in E2 suppression between AG and RG 800 mg bd was not statistically significant ($P$-value $>0.05$ Wilcoxon).

The mean (E3 + E1) % aromatisation values and their % inhibition are shown individually for each patient for AG and RG in Tables II and IV respectively. The mean ± s.e.m. % inhibition is displayed in Figures 1 and 2 respectively. Patients treated with AG show a high level of aromatase inhibition: 90.6% ± 1.8 s.e.m. The patients treated with RG demonstrated a dose response relationship for aromatase inhibition: rising from 50.6% ± 9.8 s.e.m. at 200 mg bd to a maximal suppression of 73.8% ± s.e.m. at 800 mg bd. Statistical significance was achieved ($P$-value $<0.025$ Friedman) but it was not possible to demonstrate any further significance between doses because of limited numbers of observations. Patient RB had the highest aromatase inhibition (90%) on treatment with RG. This was seen at 200 mg bd of RG with slight variations at 400 mg bd (75.6%) and 800 mg bd (86%). Patient EH exhibited a higher inhibition rate at 400 mg bd than 800 mg bd, the reason for this is unclear. RG aromatase inhibition rates showed wide interpatient variation at 200 mg bd (14.7–89.9%) and 400 mg bd (47.7–75.7%). Only at the 800 mg bd dose did the range fall (71.1–86.6%) corresponding to the narrower range seen with AG (84.6–97.8%). There was no correlation in the degree of aromatase inhibition to the magnitude of the pretreatment aromatase value for either RG or AG. AG caused significantly better aromatase inhibition than RG 800 mg bd ($P<0.01$ Wilcoxon).

On treatment with both AG and RG there was a consistent increase in the [4-$^{14}$C] $E_3$:$E_1$ ratios for each individual (Table VI). Figure 3 shows the individual patient ratio increases on a log scale (used for clarity as many of the data points were superimposed). Similar individual increases were seen in the [6,7 $^3$H] $E_3$:$E_1$ ratios (not shown) except for three individuals, two on AG and one on RG, who failed to match their [4-$^{14}$C] $E_3$:$E_1$ ratios, having a negative value. This substantially lowered the median [6,7 $^3$H] $E_3$:$E_1$ values. For AG and RG 800 mg bd on-treatment the median increase in the [4-$^{14}$C] $E_3$:$E_1$ ratio was 213% (range 45–1584%) and 212% (93–1085%) and in the [6,7 $^3$H] $E_3$:$E_1$ ratio 63% (−79–1129%) and 122% (−29–1463%) respectively. For RG the ratio increase was dose related. There was a wide interpatient variation in the on-treatment and pre-treatment ratios for both AG and RG. For neither treatments was there a correlation between initial ratio value and the magnitude of increase.

**Figure 1** Mean (±s.e.m.) % inhibition of aromatase and suppression of oestradiol by Aminoglutethimide.

**Figure 2** Mean (±s.e.m.) % inhibition of aromatase and suppression of oestradiol by Rogletimide.

**Table II** Percentage inhibition of aromatase for Aminoglutethimide

| | *% Aromatisation* | | |
|---|---|---|---|
| | *Pre* | *On* | *% Inhibition* |
| RM | 1.42 | 0.08 | 89.9% |
| EM | 1.24 | 0.12 | 97.8% |
| AS | 1.34 | 0.03 | 94.1% |
| EL | 1.54 | 0.24 | 92.4% |
| CS | 0.98 | 0.10 | 84.6% |
| CD | 2.70 | 0.20 | 84.8% |
| ED | 2.49 | 0.38 | 90.5% |
| | | Mean | 90.56% ± 1.8 s.e.m. |

**Table III** Percentage inhibition of oestradiol for Aminoglutethimide

| | *Oestradiol* | | |
|---|---|---|---|
| | *Pre* | *On* | *% Inhibition* |
| RM | 30.6 | 4.5 | 85.3% |
| EM | 16.60 | 2.8 | 83.1% |
| AS | 14.4 | 4.7 | 67.4% |
| EL | 34.0 | 22.0 | 35.2% |
| CS | 37.7 | 6.4 | 83.0% |
| CD | 46.0 | 4.1 | 91.1% |
| ED | 34.4 | 5.5 | 94.0% |
| | | Mean | 75.7% ± 7.3 s.e.m. |

In small studies analysis of patient response is not possible but merits comment. Patients were formally staged at the start of the study and at 3 months for AG or completion of the study for RG. There was one responder (UICC criteria) in the AG group. It was encouraging to note the prolonged disease stable treatment periods with RG (21 months and 18 months, patient EG and RB respectively), in view of its suboptimal E2 suppression and aromatase inhibition, in a group of patients with advanced breast cancer and limited life expectancy. Toxicity necessitating dose reduction was experienced by only one patient (KD) and this was with RG 800 mg bd. No long term toxicity has been noted.

### Discussion

Any variations in E2 suppression and aromatase inhibition between AG and RG are difficult to explain in terms of

**Table IV** Percentage inhibition of aromatase for rogletimide

| | *Pre* | *200 mg bd* | *% inhibition* | *400 mg bd* | *% inhibition* | *800 mg bd* | *% inhibition* |
|---|---|---|---|---|---|---|---|
| EG | 2.91 | 1.52 | 47.7% | 0.71 | 75.8% | 0.47 | 84.0% |
| JL | 0.68 | 0.58 | 14.7% | 0.36 | 47.7% | 0.20 | 71.1% |
| MM | 1.13 | 0.51 | 55.0% | 0.35 | 69.1% | 0.30 | 73.2% |
| RB | 2.93 | 0.30 | 89.9% | 0.71 | 75.6% | 0.39 | 86.6% |
| EH | 1.29 | 0.74 | 42.6% | 0.41 | 68.2% | 0.61 | 53.8% |
| KD | 0.43 | 0.20 | 53.4% | 0.24 | 44.0% | N/A | |
| | | Mean | 50.6% ± 9.8 s.e.m. | | 63.5% ± 5.7 s.e.m. | | 73.8% ± 5.8 s.e.m. |

**Table V** Percentage inhibition of oestradiol for rogletimide

| | *Pre* | *200 mg bd* | *% inhibition* | *400 mg bd* | *% inhibition* | *800 mg bd* | *% inhibition* |
|---|---|---|---|---|---|---|---|
| EG | 10.0 | 9.1 | 10.0% | 7.7 | 20.0% | 7.1 | 23.0% |
| JL | 8.8 | 5.3 | 39.7% | 7.7 | 12.5% | 5.0 | 44.2% |
| MM | 42.0 | 32.0 | 23.9% | 14.0 | 66.7% | 6.5 | 84.5% |
| RB | 42.0 | 18.0 | 58.2% | 19.0 | 55.8% | 9.3 | 77.9% |
| EH | 13.0 | 15.0 | 0% | 7.3 | 44.9% | 5.9 | 61.5% |
| KD | 12.0 | 5.9 | 51.9% | 5.6 | 54.4% | N/A | |
| | | Mean | 30.7% ± 9.5 s.e.m. | | 40.2% ± 10.3 s.e.m. | | 57.6% ± 9.2 s.e.m. |

**Figure 3** Individual patient ratios of (4-$^{14}$C) $E_3$:$E_1$ with Aminoglutethimide (AG) and Rogletimide (RG).

patient selection, or previous treatments, as all demographic parameters were similar.

The pre-treatment aromatase values and plasma E2 levels for all patients fell within previously described ranges (Santen *et al.*, 1978; Jacobs *et al.*, 1992). The percentage E2 suppression for RG is consistent with previous work (Dowsett *et al.*, 1991; Haynes *et al.*, 1991). The difference in E2 suppression between the two drugs did not achieve statistical significance. This may be the result of small patient numbers or assay limited sensitivity making individual values uncertain. Aromatase inhibitors suppress the already low post-menopausal plasma E2 levels close to assay sensitivity limits which may be relevant in concealing further suppression. The lowest E2 levels seen for AG (2.8 pmol $l^{-1}$) are at the limit of assay sensitivity. Those for RG (5.0 pmol $l^{-1}$) were not. This supports the difference between RG and AG in E2 suppression as being real. The degree of E2 suppression by RG is comparable to that seen with the other aromatase inhibitors currently under investigation (Dowsett *et al.*, 1989, 1990). In this study AG E2 suppression is at the top end of that previously reported (Harris *et al.*, 1982; Santen *et al.*, 1978, 1982; Vermeulan *et al.*, 1983). *In vivo* tracer measurements of aromatase activity are a more sensitive means of discriminating between different drug schedule influences on oestrogen suppression (Lonning *et al.*, 1991). The isotopic tracer studies showed a maximal aromatase inhibition of 91% for AG 1,000 mg daily. This value is lower than the 98% previously reported (Santen *et al.*, 1978) but used different methodology which may allow more accurate quantification of aromatase conversion rates (Jacobs *et al.*, 1991). It corresponds well to the maximal aromatase inhibition rates seen with other novel aromatase inhibitors we have investigated in this manner: CGS 16949A 2 mg bd (Lonning *et al.*, 1991) and 4-hydroxyandrostenedione (4-OHA) 500 mg intramuscularly fortnightly (Jones *et al.*, 1992), confirming the usefulness of AG for treating postmenopausal breast cancer. It has yet to be demonstrated that the degree of aromatase inhibition correlates with clinical efficacy and it will be of interest to discover whether 4-OHA and CGS 16949A can match the clinical successes of AG.

In comparison RG 800 mg bd achieved only 74% aromatase inhibition. This further suggests that the difference in plasma E2 suppression between AG and RG reflects a true difference in pharmacological activity. RG shows a wide variation in the degree of aromatase inhibition at the lower

**Table VI** (4-$^{14}$C) E3:E1 ratios for aminoglutethimide and rogletimide

| | *Aminoglutethimide* | | |
|---|---|---|---|
| | *Pre* | *On* | *% Increase* |
| RM | 1.5 | 4.7 | 213 |
| JL | 1.1 | 2.2 | 118 |
| AS | 0.6 | 1.1 | 80 |
| EL | 0.4 | 6.4 | 1584 |
| CS | 8.5 | 28.0 | 229 |
| CD | 10.7 | 33.0 | 208 |
| ED | 9.7 | 14.1 | 45.4 |
| | Median | | 213% |
| | *Rogletimide* | | |
| | *Pre* | *On* | *% Increase* |
| EG | 0.4 | 1.3 | 237 |
| EM | 0.6 | 1.5 | 131 |
| MM | 1.7 | 3.6 | 93 |
| RB | 7.0 | 23.8 | 223 |
| EH | 0.4 | 5.0 | 1085 |
| KD | 1.0 | 3.2 | 199 |
| | Median | | 212% |

doses of 200 mg bd and 400 mg bd. Only at 800 mg bd does the large interpatient range narrow, as the degree of aromatase inhibition becomes more uniform, reflecting a more effective dosing schedule for the majority of patients. The reason for RGs' inferior aromatase inhibition is not clear but may be two-fold. (i) Poor tissue/plasma concentrations. Early work demonstrated that RG oral bioavailability, like AG, was excellent and that doses of 500 mg bd were probably supramaximal for maintaining serum levels of RG above the 2 $\mu g\ ml^{-1}$ threshold required for effective E2 suppression (Haynes *et al.*, 1991). However unpublished data from a previous study (Dowsett *et al.*, 1991) suggested that even at 800 mg bd, plasma RG trough levels were consistently below the required therapeutic threshold in 55% of patients. This contradiction may be the result of RG inducing its own metabolism similar to AG, induction only being detectable on more prolonged dosing schedules than previously studied i.e. 2 weeks compared with previous 5 days. (ii) In the early stages of RG development it was chosen from a series of analogues (Leung *et al.*, 1987) (many of whom had better *in vitro* aromatase inhibitory potential) because of its long half life and inactive, non-toxic metabolites. Consequently an adequate *in vitro* potency comparable to that of AG has not translated as well to the clinical *in vivo* setting. Toxicity limits dose escalating RG any further but one could assume that the dose/aromatase inhibition relationship would be maintained at higher doses of RG.

During this study it was noted that the E3:E1 isotope ratios in the urine changed in the on treatment situation for both AG and RG. This had not been noted in our previous in-vivo work on CGS 16949A (Lonning *et al.*, 1991) and 4-OHA (Jones *et al.*, 1992). Recent work has suggested that AG may have a dual mechanism of action in oestrogen suppression: (i) direct aromatase inhibition, (ii) stimulation of oestrogen metabolism consequent upon hepatic microsomal enzyme induction, increasing plasma E1S clearance and decreasing the E1S/E1 and E1S/E2 ratios (Lonning *et al.*, 1987, 1989*b*). The mechanism by which this occurs is stimulation of the 16 a hydroxylase enzyme (Lonning & Skulstad, 1989) which converts oestrogens to E3. Any alteration in the direction of oestrogen metabolism i.e. an increase towards E3 production and urinary excretion with a fall in that of E1 (Lonning *et al.*, 1987) will be reflected in the change of oestrogen ratios. The change in oestrogen ratios is unlikely to be an analytical artifact: (i) we have not seen this phenomenon with any other aromatase inhibitors studied in this manner. (ii) E1 and E3 losses are probably similar through out the purification and separation stages of our analysis as both steroids unlike the catechol (2-OH) oestrogens are known to be chemically stable and do not undergo oxidative degradation. If a selective analytical loss of either of these oestrogens accounted for the ratio change it would be seen in all analysis not just those performed on RG and AG. If plasma E1S is quantitively a more important source of unconjugated oestrogens than androstendione in the postmenopausal women and a potential source of oestradiol in breast tumours (Santner *et al.*, 1986), reducing the circulating storage pool of E1S may give AG important clinical advantages over other more specific aromatase inhibitors (Lonning *et al.*, 1989*a*). As an AG analogue, RG would be expected to have similar properties. This hypothesis is indirectly supported by the comparable changes seen in the 14C E3:E1 isotope ratios on RG and AG treatment. 14C E3:E1 ratio reversal similar to AG was seen at 800 mg bd of RG, indicating that comparable hepatic enzyme induction may be achieved at this dose of RG, even if aromatase inhibition is not. The wide interpatient variations in pre and on-treatment E3:E1 isotope ratios for both AG and RG is the result of unknown and known factors influencing hepatic enzyme function, e.g. diet, alcohol, drugs thyroid hormones. The differences seen between the 3H and 14C E3:E1 ratios in three patients in the on-treatment situation is difficult to explain. If, as the above results suggest, liver microsomal enzyme induction on RG is comparable to AG, then RG's suboptimal E2 suppression may be totally accounted for by inferior aromatase inhibition.

The difficulties of relying only on endocrine parameters for aromatase inhibitor assessment is underlined in the work presented here, for although the degree of E2 suppression for RG is lower than AG, which is consistent with its lower aromatase inhibition rates, it was not statistically significant. Until we can find or refute a link between oestrogen suppression and tumour response we can persue and investigate only those aromatase inhibitors deemed most effective pharmacologically by full aromatase evaluation.

### Conclusion

At the maximum tolerated dose of 800 mg bd, RG achieves inferior E2 suppression and aromatase inhibition, compared with its parent compound AG. Whether this is the result of inadequate tissue/plasma drug concentration or poor aromatase inhibitory potency is of no clinical relevance as further dose escalation of RG is limited by toxicity. It is difficult to envisage a role for RG in the management of advanced breast cancer in view of newer, more pharmacologically effective aromatase inhibitors with minimal toxicity, now being developed. *In vivo* aromatisation measurements performed in conjunction with plasma oestrogen analysis creates a powerful tool for evaluating the optimal therapeutic dose and potential clinical efficacy of any new aromatase inhibitors in small groups of patients. Early evaluation employing these methods will select out aromatase inhibitors with low therapeutic potential reducing the need for multiple large scale clinical trials to assess clinical potential.

### References

DOWSETT, M., GOSS, P., POWLES, T.J., BRODIE, A. & JEFFCOATE,S. (1987). Use of the aromatase inhibitor 4-hydroxyandrostenedione in postmenopausal breast cancer: Optimization of therapeutic dose and route. *Cancer Res.*, **47**, 1957.

DOWSETT, M., CUNNINGHAM, D.C., STEIN, R. & 4 others (1989). Dose related endocrine effects and pharmacokinetics of oral and intramuscular 4-hydroxyandrostenedione in postmenopausal breast cancer patients. *Cancer Res.*, **49**, 1306.

DOWSETT, M., STEIN, R.C., MEHTA, A. & COOMBES, R.C. (1990). Potency and selectivity of the non-steroidal aromatase inhibitor CGS16949A in postmenopausal women with breast cancer. *Clin. Endocrinol.*, **32**, 623.

DOWSETT, M., MACNEILL, F.A., MEHTA, A. & 7 others (1991). Endocrine, pharmacokinetic and clinical studies of the aromatase inhibitor 3-ethyl-3(4-pyridyl)piperidine-2,6-dione ('pyridoglutethimide') in postmenopausal breast cancer patients. *Br. J. Cancer*, **64**, 887.

FOSTER, A.B., JARMAN, M., LEUNG, C.S., ROWLANDS, M.G. & TAYLOR, G.N. (1985). Analogues of Aminoglutethimide: selective inhibition of aromatase. *J. Med. Chem.*, **28**, 200.

GOSS, P.E., POWLES, T.J., DOWSETT, M. & 4 others (1986). Treatment of advanced postmenopausal breast cancer with an aromatase inhibitor, 4-hydroxyandrostenedione: phase II report. *Cancer Res.*, **46**, 4823.

GRODIN, J.M., SIITERI, P.K. & MACDONALD, P.C. (1973). Source of oestrogen production in postmenopausal women. *Journal Clin. Endocrinol. Metab.*, **36**, 207.

HALL, T., BARLOW, J., GRIFFITHS, C. & SABA, Z. (1969). Treatment of metastatic breast cancer with Aminoglutethimide. *Clin. Res.*, **17**, 402.

HARRIS, A.L., POWLES, T.J. & SMITH, I.E. (1982). Aminoglutethimide in the treatment of advanced post menopausal breast cancer. *Cancer Res.*, Suppl. 42, 3405.

HARRIS, A.L., CANTWELL, B.M.J. & DOWSETT, M. (1988). High dose Ketokonozole: Endocrine and therapeutic effects in the postmenopausal breast cancer women. *Br. J. Cancer*, **58**, 493.

HAYNES, B.P., JARMAN, M. DOWSETT, M. & 5 others (1991). Pharmacokinetics and pharmacodynamics of the aromatase inhibitor 3-ethyl-3(4-pyridyl)piperidine-2,6-dione in patients with postmenopausal breast cancer. *Cancer Chemother. Pharmacol.*, **27**, 367.

JACOBS, S., LONNING, P.E., HAYNES, B., GRIGGS, L. & DOWSETT, M. (1991). Measurement of aromatisation by a urine technique suitable for the evaluation of aromatase inhibitors *in vivo*. *J. Enzyme Inhib.*, **4**, 315.

JACOBS, S., MACNEILL, F.A., LONNING, P., JONES, A., DOWSETT, M. & POWLES, T.P. (1992). Aromatase activity, plasma oestrogens and their correlation with demographic indices in postmenopausal women with breast cancer. *J. Steroid Biochem.*, **41**, 769.

JONES, A.L., MACNEILL, F.A., JACOBS, S., LONNING, P., POWLES, T.J. & DOWSETT, M. (1992). The influence of intramuscular 4-hydroxyandrostenedione on peripheral aromatisation in breast cancer patients. *Eur. J. Cancer*, (in press).

LEUNG, C.S., ROWLANDS, M.C., JARMAN, M., FOSTER, A.B., GRIGGS, L.J. & WILMAN, D.E.V. (1987) Analogues of 3-ethyl-3-(4-pyridyl)piperidine-2,6-dione as selective inhibitors of aromatase: derivitives with variable 1-alkyl and 3-alkyl substituents. *J. Med. Chem.*, **30**, 1550.

LONNING, P.E., KVINNSLAND, S., THORSEN, T. & UELAND, P. (1987). Alterations in the metabolism of oestrogens during treatment with Aminoglutethimide in breast cancer patients: Preliminary findings. *Clin. Pharmacokinet.*, **13**, 393.

LONNING, P.E. & KVINNSLAND, S. (1988). Mechanisms of action of Aminoglutethimide as an endocrine therapy of breast cancer. *Drugs*, **35**, 685.

LONNING, P.E., JOHANNESSEN, D.C. & THORSEN, T. (1989*a*). Alterations in the production rates and the metabolism of oestrone and oestrone sulphate in breast cancer patients treated with Aminoglutethimide. *Br. J. Cancer*, **60**, 170.

LONNING, P.E., JOHANNESSEN, D.C., THORSEN, T. & ESKE, D. (1989*b*). Effects of Aminoglutethimide on plasma oestrone sulphate not caused by aromatase inhibition. *J. Steroid Biochem.*, **33**, 541.

LONNING, P.E. & SKULSTAD, P. (1989). Alterations in the urine excretion of Oestrogen metabolites in breast cancer women treated with Aminoglutethimide. *J. Steroid Biochem.*, **33**, 565.

LONNING, P.E., JACOBS, S., JONES, A., HAYNES, B., POWLES, T.J. & DOWSETT, M. (1991). The influence of CGS16949A on peripheral aromatisation in breast cancer patients. *Br. J. Cancer*, **63**, 789.

SANTEN, R.J., SANTNER, S., DAVIS, B. & 4 others (1978). Aminoglutethimide inhibits extraglandular oestrogen production in postmenopausal women with breast cancer. *J. Clin. Endocrinol. Metab.*, **47**, 1257.

SANTEN, R.J., SAMOJLIKE, E. & WORGUL, T. (1981). In *A comprehensive Guide to the Therapeutic use of Aminoglutethimide*. Santen, R.J. & Henderson, I.C. (eds) 101. Karger, Basel.

SANTEN, R.J.,WORGUL, T.J., LIPTON, A. & 4 others (1982). Aminoglutethimide in the treatment of postmenopausal women with advanced breast cancer. *Ann. Intern Med.*, **96**, 94.

SANTNER, S.J., LESZCZYNSKI, D., WRIGHT, C., MANNI, A., FEIL, D. & SANTEN, R.J. (1986). Oestrone sulphate: a potential source of oestradiol in human breast cancer tissue. *Breast Cancer Res. & Treat.*, **7**, 35.

STEIN, R.C., DOWSETT, M., HEDLEY, A. & COOMBES, R.C. (1990*a*). Preliminary study of treatment of advanced breast cancer in postmenopausal women with the aromatase inhibitor CGS-16949A. *Cancer Res.*, **50**, 1381.

STEIN, R.C., DOWSETT, M., HEDLEY, A., GAZET, J.C., FORD, H.T. & COOMBES, R.C. (1990*b*). Treatment of breast cancer in postmenopausal women with 4-hydroxyandrostenedione. *Cancer Chemoth. Pharmac.*, **26**, 75.

STOLL, B.A. (1981). Breast cancer rationale for endocrine therapy. In *Hormone Management of Endocrine Related Cancer*. Stoll, B.A. (ed.) 77. LLoyd Luke: London.

VERMEULAN, A., PARIDEANS, R. & HEUSON, J.C. (1983). Effects of Aminoglutethimide on adrenal steroid secretion. *J. Clin. Endocr.*, **29**, 673.